# Probabilistic reconstruction of the tumor progression process in gene regulatory networks in the presence of uncertainty

**DOI:** 10.1186/1471-2105-12-S10-S9

**Published:** 2011-10-18

**Authors:** Mohammad Shahrokh Esfahani, Byung-Jun Yoon, Edward R Dougherty

**Affiliations:** 1 Department of Electrical and Computer Engineering, Texas A&M University, College Station, Texas, USA; 2 Computational Biology Division, Translational Genomics Research Institute (TGen), Phoenix, Arizona, USA

## Abstract

**Background:**

Accumulation of gene mutations in cells is known to be responsible for tumor progression, driving it from benign states to malignant states. However, previous studies have shown that the detailed sequence of gene mutations, or the steps in tumor progression, may vary from tumor to tumor, making it difficult to infer the exact path that a given type of tumor may have taken.

**Results:**

In this paper, we propose an effective probabilistic algorithm for reconstructing the tumor progression process based on partial knowledge of the underlying gene regulatory network and the steady state distribution of the gene expression values in a given tumor. We take the BNp (Boolean networks with pertubation) framework to model the gene regulatory networks. We assume that the true network is not exactly known but we are given an uncertainty class of networks that contains the true network. This network uncertainty class arises from our partial knowledge of the true network, typically represented as a set of local pathways that are embedded in the global network. Given the SSD of the cancerous network, we aim to simultaneously identify the true normal (healthy) network and the set of gene mutations that drove the network into the cancerous state. This is achieved by analyzing the effect of gene mutation on the SSD of a gene regulatory network. At each step, the proposed algorithm reduces the uncertainty class by keeping only those networks whose SSDs get close enough to the cancerous SSD as a result of additional gene mutation. These steps are repeated until we can find the best candidate for the true network and the most probable path of tumor progression.

**Conclusions:**

Simulation results based on both synthetic networks and networks constructed from actual pathway knowledge show that the proposed algorithm can identify the normal network and the actual path of tumor progression with high probability. The algorithm is also robust to model mismatch and allows us to control the trade-off between efficiency and accuracy.

## Background

The construction of gene regulatory networks is an extremely difficult problem owing to their complexity and the difficulty of obtaining the relevant time series data, in terms of sampling rate, measurement accuracy, and quantity. For instance, microarray data usually come in samples much too small for accurate inference, have a very low sampling rate relative to most cell signaling, measure average transcript across many cells, and are susceptible to many confounding factors which adversely affect the signal-to-noise ratio. In particular, for human cells, with data coming from patients, there are no time-course data and the data come from cells that have already undergone a sequence of mutations, so that the regulatory mechanisms of the original cell are no longer intact. Rather than depend on expression data, one can use known pathway information to construct regulatory relations and thereby develop an *uncertainty class of networks* whose regulatory dynamics are consistent with the pathway knowledge. An algorithm for doing this has been developed in the context of Boolean networks [1]. If one could obtain wild-type time-course data, then one could reduce this uncertainty class by standard Boolean network inference methods. Given that in practice we usually only have access to stationary patient data and that the progression of mutations leading to the cancerous state has already occurred, we would like to use the available data to reduce the uncertainty class. In fact, since all we require is that we have an uncertainty class to begin with and wish to use the tumor data, from an algorithmic perspective it does not matter whether the uncertainty class arises from prior biological knowledge, wild-type data, or a combination of both. The proposed algorithm operates on the basis of probabilistic sequential fault-detection, which views regulatory alterations, such as gene mutations, as faults in the network wiring [2]. It estimates the sequence of faults leading to the current (cancerous) regulatory structure, and from these estimates, a reduced uncertainty class for the original (healthy) network. By taking this approach the algorithm simultaneously accomplishes a dual purpose: *network inference and fault detection*.

The methodology is based on certain fundamental notions regarding cancer development, in particular, that the formation of a tumor is a complex process usually proceeding over a period of decades. Normal cells evolve into cells with increasingly neoplastic phenotypes. Tumor progression is driven by a sequence of randomly occurring mutations and epigenetic alterations of DNA that affect the genes controlling cell proliferation, survival, and other traits associated with malignant cell phenotype. To wit, tumor progression is a multi-step process of changes in the regulatory pathways. A set of pathways must be deregulated during the tumor progression until the tissue reaches a cancer state. A wide variety of normal adult human cell types can be transformed experimentally by perturbing five pathways [3]. Certain normal human cells require a greater or lesser number of changes before they will become transformed. Moreover, the regulatory pathways can be altered in many different ways leading to the same cancer. For instance, studies on colon cancer show that the great majority (~ 90%) of colon carcinomas suffer inactivation of the *APC* gene on Chromosome 5q as an early step in this process, about 40% to 50% acquire a K-*ras* mutation, 50% to 70% show an LOH of Chromosome 17p involving p53, and about 60% show an LOH on Chromosome 18q. Most colon cancers will therefore begin with a Chromosome 5 alteration, but then will take alternative genetic paths on the road toward full-fledged malignancy [3].

In sum, although some common alterations may happen in tumor progression, different patients confront with different types of alterations during the progression, thereby making it important to find a way to identify mutations in order to apply appropriate intervention. Very little work has been done on the identification of genetic alterations (e.g. mutations) in cancer progression using network models. One such example is the work by Gerstung et al. [4], where they predicted cancer progression by applying a conjunctive Bayesian network, in which the order of gene mutations is extracted.

In this paper, we use the Boolean *networks with perturbation* (BNp) framework to model signaling pathways and ultimately predict the gene mutations that occurred during the tumor progression process. Boolean networks (BNs) have been used in a variety of other contexts and with different objectives in biological applications. Kauffman [5] proposed that the cell types are the attractors. He introduced randomization into the networks, in terms of environmental noise (random perturbation of individual genes) and mutation (not to be confused with the notion of mutation in cancer progression), which refers to changes in the wiring of the network. Random BNs and their characteristics have been extensively studied by Aldana et. al [6]. In a random BN, the average function in-degrees are constant and function outputs are assigned randomly. Serra et al. [7] investigated the effects of perturbation in the context of random BNs by knocking out a single gene. Additionally, intervention in BNp has been also studied by Dougherty et al. [8] and Qian and Dougherty [9].

In this work, we focus on BNs with perturbation owing to their role in modeling gene regulatory networks, a key point being that their dynamics can be modeled as Markov chains, thereby facilitating the modeling of genetic alterations in signaling pathways by shifting the network steady state distribution (SSD) from the normal (healthy) SSD toward the cancerous SSD. Having this tool in one hand to model signaling pathways, and the cancerous SSD extracted from the malignant tissue (e.g., based on gene expression data) on the other hand, one can test all the possible alterations on the BN satisfying the pathway information to see which one makes the SSD of the altered BN as close to the cancerous SSD as possible. This allows one to track the sequence of mutations. However, there are two concerns for using BNps to model signaling pathways: (1) the network perturbation probability should be determined, and (2) signaling pathways provide us with incomplete information, which means that there may be too many BNs that satisfy the pathway information. The first issue can be mitigated by finding a good estimate of the perturbation probability. For example, inferring a BNp from a sequence of gene expression data has been studied in [10]. In fact, the second issue is the main source of uncertainty in our problem. To the best of our knowledge, the paper by Layek et al. [1] is the only work that proposed a method to extract the BN underlying the normal tissue from a set of biological pathways. Although this paper introduced an elegant method for extracting the information needed for constructing Boolean networks from biological pathways, it yields a large number of networks since the available network knowledge is often incomplete and not enough to point out the true network. To address this issue, we define the notion of a *family of Boolean networks*, which is the set of all BNs that satisfy the constraints that are imposed by a given set of pathways. For instance, for a 6-gene signaling pathway, the resulting family can contain 2^12^ networks, all of which satisfy the constraints imposed by the pathway.

As mentioned earlier, the main goal of this paper is twofold: (1) to infer the normal network underlying healthy cells from this family, and at the same time, (2) to find the set of alterations that have occurred during the cancer progression. Toward this goal, we propose a probabilistic sequential fault detection algorithm that can effectively identify the best candidates for the original normal (healthy) network and the accumulated gene mutations.

## Methods

### Boolean networks (BNs)

A Boolean network *G* (*V*, *F*) is defined by a set *V* = {*x*_1_, *x*_2_…,, *x_n_*} of binary variables, *x_i_* ∈ {0,1}, *i* = 1,…, *n*, and a list *F* = (*f*_1_, *f*_2_,…, *f_n_*) of Boolean functions. The value of *x_i_* at time *t* + 1 is completely determined by a subset {*x_i_*_1_, *x_i_*_2_, … ,*x_i_k_i___*} ⊂ *V* at time *t* via a Boolean function *f_i_* : {0, 1}*^k_i_^ →* {0,1}. Transitions are homogeneous in time and we have the update:(1)

Each *x_i_* represents the state (expression) of gene *i*, where *x_i_* = 1 and *x_i_* = 0 represent gene *i* being expressed and not expressed, respectively. It is commonplace to refer to *x_i_* as the *i*th gene. The list *F* of Boolean functions represents the rules of regulatory interactions between genes. That is, any given gene transforms its inputs (regulatory factors that bind to it) into an output, which is the state or expression of the gene itself. All genes are assumed to update synchronously in accordance with the functions assigned to them and this process is then repeated. At any time *t*, the state of the network is defined by a state vector **x**(*t*) = (*x*_1_(*t*), *x*_2_(*t*), *…*,*x_n_*(*t*)), called a *gene activity profile* (GAP). Given an initial state, a BN will eventually reach a set of states, called an *attractor cycle*, through which it will cycle endlessly. Each initial state corresponds to a unique attractor cycle and the set of states leading to a specific attractor cycle is known as the *basin of attraction* (BOA) of the attractor cycle.

A *Boolean network with perturbation* (BNp) is defined by allowing each gene to possess the possibility of randomly flipping its value with a positive probability *p*. Implicitly, we assume that there is an i.i.d. random perturbation vector γ = (γ_1_, γ_2_, …, γ_n_), where γ_i_ ∈ {0, 1}, the *i*th gene flips if and only if γ*_i_* = 1, and *p* = *P*(*γ_i_* = 1) for *i* = 1, 2,…, *n*. If **x**(*t*) is the GAP at time *t*, then the next state **x**(*t* + 1) is either **f**(**x**(*t*)) with probability (1 – *p*)*^n^* or **x**(t) ⊕ γ with probability 1 – (1 *–p*)*^n^*, where **f** is the multi-output function from the truth table and ⊕ is component-wise addition modulo 2. Perturbation makes the corresponding Markov chain of a BNp irreducible and ergodic. Hence, the network possesses a steady state distribution, *SSD*(*BNp*), describing its long-run behavior. A BNp inherits the attractor structure from the original Boolean network without perturbation, the difference being that a random perturbation can cause a BNp to jump out of an attractor cycle, perhaps then transitioning to a different attractor cycle prior to another perturbation. If *p* is sufficiently small, then the SSD will reflect the attractor structure within the original network. We can derive the transition probability matrix (TPM) **P** if we know the truth table and the perturbation probability *p* for a BNp. The TPM of a BNp can be decomposed as:(2)

where, **Q** is the TPM of the corresponding deterministic BN and **H** is a 2*^n^* × 2*^n^* matrix depending only on *n* and *p *[11].

We assume there exists a “normal” BN, denoted *N_normal_*, corresponding to a healthy wild-type phenotype, and a family  of BNs possessing identical predictor sets as *N_normal_* such that . We refer to this family  as the “uncertainty class” relative to *N_normal_*.

Given a BN, we define an “alteration” to be a change in the rule structure (i.e., truth table). A “path” *Path* = {*alt*_1_ , *alt*_2_, …, *alt_M_*} is defined as a sequence of *M* alterations. From a modeling perspective, *M* denotes the number of alterations that have occurred during the tumor progression and *alt_j_* refers to the *j*th alteration. We assume that each alteration affects only a single gene and no two alterations in the same path affect the same gene. The result of applying a path of alterations to a Boolean network *N* is to produce an “altered network” [*N; Path*]. If we begin with a normal BN, *N_normal_*, and apply a “cancerous path”, *Path_c_*, we obtain a cancerous network *N_cancer_* =[*N_normal_; Path_c_*]. The following commutativity and associativity properties follow from the definition:(3)

Alterations in cancer progression are commonly gene mutations, and the accumulation of gene mutations is usually responsible for cancer. Gene mutation includes both *oncogene activation* and *tumor suppressor gene deactivation*, resulting in either continuous activation or deactivation of the corresponding genes. In the context of the BN model, such alteration in gene *x_i_* leads to permanently setting the boolean function to *f_i_* ≡ 1 or *f_i_* ≡ 0. We denote a gene mutation by a pair (*i*, k), which indicates that gene *x_i_* is stuck at *k* ∈ {0, 1}. For convenience, we define  and . If a Boolean network *N* is altered by a mutation (*i*, *k*), this mutated BN is denoted as [*N;* {(*i*, *k*)}]. For example, for a 4-gene Boolean network *N*, [*N;* {(1,0), (4,1)}] refers to a mutated version of *N*, where gene *x*_1_ is permanently deactivated and gene *x*_4_ is permanently activated. In this case, in the regulatory truth-table, we will have *f*_1_ = 0 and *f*_4_ = 1 for every set of predictors. Gene mutation, also called “1-gene function perturbation”, has been studied [9]. It should be noted that, physically, the order of mutations can make a difference in cancer progression, since alterations affect the regulatory structure, thereby affecting subsequent cancer progression. There is, however, no way to take this into account given that we only have steady-state data and no data on transient behavior. From a mathematical perspective, the commutativity in (3) means that a path is a *set* of alterations rather than a *sequence* of alterations; however, we employ the latter terminology owing to its commonplace usage.

Now the problem to be addressed in this paper can be stated as follows: Given a family  of Boolean networks, the steady state distribution (SSD) of the cancerous network, and the number of alterations *M*, what are the best candidates for *N_normal_* and *Path_c_?* Searching for the best candidate for the normal network involves estimating the distance between altered networks and the cancerous network. Since the only available information about the cancerous network is its SSD, we need to define a distance measure between two networks based on their SSDs. Given two BNs with perturbation  and , we compute their distance as follows:(4)

where , , and *ρ*(π_i_,π_j_) is the Kullback-Leibler divergence (KL-divergence) between the SSDs π*_i_* and π*_j_*. This distance measure can be extended to BNs by first building the corresponding BNp for each BN using (2) and a given probability of perturbation *p* and then computing the distance between the resulting BNps. Without any ambiguity, in what follows, we use the same notation for a BN and the induced BNp for notational simplicity.

### Gene mutation effects

#### Effects of gene mutation in a BNp

In this section, we study the effect of a gene mutation (*i*, *k*) on the TPM of a BNp and its SSD. Gene mutations affect only the regulatory matrix **Q** in (2), where the mutation of each gene can be modeled as a multiplicative perturbation. Thus, for every mutation (*i*, k), we can find a corresponding *transformation matrix***T**_*i*,*k*_ such that the TPM of the altered BNp is given by:(5)

where **I** is a 2*^n^* × 2*^n^* identity matrix. Based on this observation, we can easily prove the commutativity property shown in (3) (see Additional file 1 for the proof). According to the associative property shown in (3), a sequence of multiple gene mutations can be represented by a single transformation matrix, which is a product of the transformation matrices, each corresponding to a single gene mutation in the sequence. For example, the TPM of a BNp altered by a threefold mutation, [*N;* {(*i*_1_, *k*_1_), (*i*_2_, *k*_2_), (*i*_3_, *k*_3_)}], is given by:

The effect of rank-one perturbations in the TPM of a Markov chain on the SSD has been studied in the context of structural intervention in gene regulatory networks [9], and more generally in the framework of Markov chain perturbation theory [12]. We can utilize these results to analyze the SSD of the altered BNp, whose TPM is given by (5).

In order to see how existing work on Markov chain perturbation can be used to analyze the effect of gene mutations on the SSD, consider two TPMs **P** and  that arise from the original network and the altered network, respectively. Let π and  be the SSDs of the two networks, such that π*^τ^***P** = *π^τ^* and . We can find the analytical expression of the change in SSD using the fundamental matrix **Z** = [**I** – **P** + eπ^τ^]^–1^, where e is an all-one column vector [13]. The fundamental matrix **Z** exists for any ergodic Markov chain. Consider a *rank-one perturbation*, where the TPM of the perturbed Markov chain is , where **a**, **b** are two arbitrary vectors satisfying **b**^τ^**e** = 0, and **P** is the TPM of the original Markov chain. In this case, it can be shown that [14] the following is true:(6)

Now, by representing the change of TPM due to a gene alteration as a sequence of rank-one perturbations, we can utilize (6) to predict the overall effect of the given mutation on the SSD of the network. To be more precise, suppose the BNp at hand undergoes a single mutation, (*i*, *k*). The transition probability matrix  of the mutated BNp can be represented as follows:(7)

for some vectors **a***_j_* and **b***j* satisfying , and a positive integer *u* ≤ 2^n – 1^. The proof can be found in Additional file 1. Based on (6) and (7), the SSD of the altered BNp can be iteratively calculated in at most 2*^n^*^–1^ iterations.

#### Example: effect of mutations in a 3-gene network

For illustration, let us consider a simple 3-gene BNp. Suppose the BNp is altered by (3,0), which means that the gene *x*_3_ is permanently deactivated such that *x*_3_ = 0. As a consequence, there cannot be any destination state in **Q**, which is the deterministic part of the TPM **P** in that arises from the regulatory structure of the BN, that corresponds to *x*_3_ = 1. Hence, if we let **Q** = [**q**_1_… **q**_8_ ], where **q***_j_* is the *jth* column in **Q**, the corresponding columns in **Q** should be shifted as follows:

where **q***_j_* corresponds to the destination state with decimal representation *j* – 1, and **q***_j_* ←**q***_i_* means the *jth* column should be updated to **q***_i_* + **q***_j_* and the *i*th column to 0. Therefore, we get the following transformation matrix:

and we have:(8)

Note that the rank of **Q**(**T**_3,0_ – **I**) is at most 2^(^*^n^*^–1)^= 4. Now, (8) can be decomposed as follows:(9)

where  for all **b***_i_*. From (9) and (5), we get:(10)

which is in the form shown in (7). Now, by utilizing the result in (6), we can analytically compute  through sequential rank-one perturbations as follows:(11)

where π and  are the SSD vectors for **P** and , respectively, which satisfy *π^T^***P** = *π^T^* and . **Z***_j_* are the corresponding fundamental matrices, as defined earlier.

### Overview of the proposed algorithm

**Figure 1:**
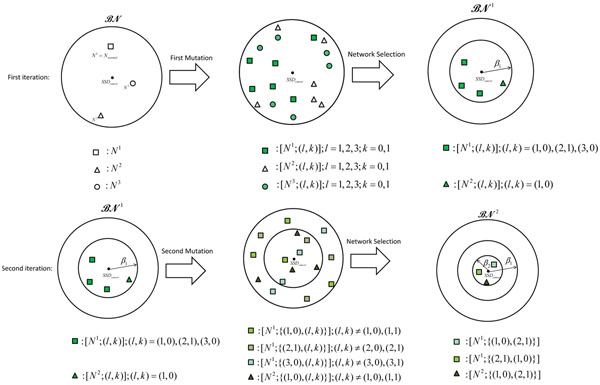
**Illustrative overview of the algorithm**. Sequential fault detection algorithm for a family  that consists of three 3-gene Boolean networks (depicted as a square, triangle, or circle). Suppose *N^1^* is the (unknown) normal network that was altered into the cancerous network through *M* = 2 mutations. In the first row, all possible single mutations are applied to all networks, where the resulting altered networks are shown in the middle. The algorithm keeps only those networks whose residual value (the distance to the cancerous network) is less than *β*_1_, resulting in a reduced family . In the next step, we consider all possible single gene mutations to the networks in , as shown in the middle of second row. The algorithm keeps only those networks whose residual value is less than *β*_2_(<*β*_1_), which leads to a further reduced set

Suppose we are given a family  of Boolean networks that contains the normal network *N_normal_*. Based on our definition, the cancerous network can be written as:

Let *SSD_cancer_* denote the SSD of the cancerous network *N_cancer_*. We define the *residual value* for a given Boolean network *N* as:(12)

where *N_p_* is the BNp with the regulatory matrix **Q** determined by the Boolean network *N* and the perturbation probability *p*. At each step, the algorithm alters the networks in the current family of networks through a single mutation. After the alterations, the algorithm keeps only those networks that lie within a certain distance from the cancerous network, where the distance is computed by (12). For the selected networks, the algorithm also keeps a record of the alterations that leads to these altered networks. Figure 1 provides an illustrative overview of the algorithm. Suppose that initially, the network family  contains three 3-gene networks. We assume that *N*^1^ is the true normal network *N_normal_*, and the cancerous network *N_cancer_* is obtained by taking the cancer progression path *Path_c_* = {(1, 0), (2,1)}, hence *N_cancer_* = [*N_normal_*; {(1,0), (2,1)}]. Given the family , the SSD of the cancerous network *SSD_cancer_*, and the number of mutations (set to *M* = 2 in this example), the algorithm tries to identify the best candidates for the normal network *N_normal_* and the path *Path_c_* that may lead the original network into the cancerous network in two steps.

Initially, for each network , there can be 6 possible altered networks based on a single gene mutation. These altered networks are shown in the first row of Figure 1, in the middle plot. Among these networks, the algorithm only selects the networks whose SSDs are close to *SSD_cancer_*. Suppose we select the altered networks that are within *β*_1_-distance of cancerous network. The selected networks constitute a new (and *reduced*) uncertainty class of networks . Next, each network in  can be altered into 4 different networks based on an additional single gene mutation. These networks are shown in the second row of Figure 1, in the middle plot. Among these networks, the algorithm selects only those that are within *β*_2_-distance from the cancerous network, resulting in a further reduced uncertainty class of networks . The family  contains the best candidates for the normal network and the cancerous path. For example,  in Figure 1 contains two candidates (*N*^1^ and *N*^2^) for the normal network. For *N*^1^, the cancerous path {(1,0), (2,1)}, and equivalently, {(2,1), (1,0)}, may lead it to the cancerous network with the given steady state distribution *SSD_cancer_*. Similarly, *N*^2^ may be another candidate for the normal network, which may get close to the cancerous network also through the path {(1,0), (2,1)}. Note that the actual number of networks in  and that in  will depend on the parameters *β*_1_ and *β*_2_, respectively.

### Details of the proposed algorithm

The detailed procedure of the proposed algorithm is shown in Algorithm 1. At each step, one additional single gene mutation is considered. Therefore, to detect all *M* alterations that may have led the normal network into the cancerous network, the algorithm needs to go through *M* sequential steps. In the first step, we consider all possible single mutations of the form (*i*, *k*) for every network *N* in the family , which results in  altered networks. Among these networks, we select only those networks whose SSD can get close enough to *SSD_cancer_*, after *M –* 1 additional gene mutations. Based on this criterion, we select the network-mutation pairs, whose residual values (distance to the cancerous network measured based on SSD) are smaller than a threshold *β*_1_. In the second iteration we start with a new family of BNs  that contains the networks selected in the previous iteration. Since the gene that was mutated in the first step cannot go through another mutation, every network in  can go through one of 2(*n* – 1) possible single gene mutations. Among these possible altered networks, we select only those networks whose residual values are smaller than a threshold *β*_2_. After repeating these steps *M* times, the final class  will contain the best network-path pairs, where each pair consists of a candidate for the normal network and the cancerous path that may drive the given network into the cancerous state with the given SSD. In each iteration, the threshold *β_j_* can be used as a control parameter for trading between efficiency and accuracy. In general, we will have *β*_1_ ≥ *β*_2_ ≥ … ≥ *β_M_*.

#### Performance metrics

In order to evaluate the performance of the algorithm, we define two metrics. The first metric is the probability that the algorithm will miss the true normal network *N_normal_* and the actual cancerous path *Path_c_* of length *M:*(13)

We can estimate this probability as follows. Let us define:(14)

where *F_D_*^(*M* – *i*,*p_cancer_*)^(*d*) is the cumulative distribution function (CDF) of the distance *d* between a BNp (with the perturbation probability *p_cancer_*) and its altered version obtained by (M – *i*) mutations. Estimation of this CDF will be further discussed in the next section. Now, if we define:(15)

we can show that:(16)

The proof can be found in Additional file 1. The second metric to be used is the probability that the algorithm will not be able to detect any network within ∈-distance of the true normal network *N_normal_* :(17)

These two metrics can be used to evaluate the accuracy of the proposed algorithm.

It would be also interesting to evaluate the computational complexity of the algorithm. When performing an exhaustive search, the total number of residual value computations that would be needed to find the final network family  would be:

which is exponential with respect to the number of mutations *M*.

Now, suppose that in the *i*th iteration of the proposed algorithm, α*_i_*% of the networks are selected (i.e.,  ) by controlling the parameter *β_i_*. For finding , our algorithm would need:(18)

residual value computations, where α_0_ = 1. A smaller *β_i_* will lead to a smaller *α_i_*, thereby reducing the overall complexity of the algorithm. However, this will also increase the probability of missing the true network, hence the parameters *β_j_* can be used to control the trade-off between computational efficiency and the prediction accuracy of the algorithm. As we can see from (18), the computational complexity of the proposed algorithm is polynomial with respect to the number of genes *n* (for a fixed M), while it is exponential with respect to the number of mutations *M* (for a fixed *n*). However, the parameters α*_j_* (*j* = 0, … , *M* – 1) allow one to trade between computational efficiency and prediction accuracy. As a result, the proposed algorithm can accurately reconstruct the cancer progression path in a much more efficient manner compared to the exhaustive search, as will be demonstrated in our simulation results.

### Cumulative distribution function of the distance between a random BNp and its mutated version

We estimate the CDF of the distance between a network and its altered version based on random BNs. We define a *random Boolean network* (RBN) as a BN: (1) whose gene predictors are randomly chosen such that every gene has *k* predictors, and (2) the truth table of every Boolean function *f_i_* follows an independent and identically distributed *Bernoulli*(*p*_b_) distribution, where *p_b_* is typically called the *bias* of the Boolean function *f_i_*. By allowing random perturbations with probability *p* in the RBN, we can obtain a random BNp (RBNp). First, we generate large number of RBNps with certain properties. Second, for each RBNp *N_p_*, we randomly introduce *m* mutations to obtain an altered network , and measure their distance . Based on these observations, we can estimate the CDF,  where *m* is the number of single gene mutations and *p* is the perturbation probability in the RBNp.

## Results and discussion

### Estimating the CDF of the distance between networks

To execute the algorithm, we first estimated the CDF  of the distance d between an RBNp and its mutated copy. As with ensemble analysis in [15][16][17], we estimate these CDFs based on a large number of randomly generated networks with similar structural properties. The two most important parameters for generating random BNs are their *bias* probability *p_b_* and *connectivityk*. As described earlier, *p_b_* is the mean of the Bernoulli distribution used to randomly generate the predictor function for each gene in a BN, and *k* is the maximum number of input variables for each of these functions. We randomly generated 4,000 BNps with these properties. For each network, we introduced random gene mutations and computed the distance between the original BNp and the altered BNp. We used the MATLAB function KSDENSITY to find the CDF that best fits the observed distance distribution. We repeated the overall experiment for different numbers of genes *n*, different perturbation probabilities *p*, and different numbers of mutations *m*.

**Figure 2:**
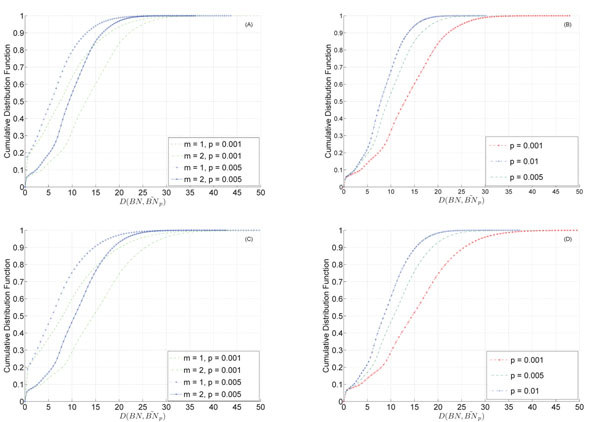
**CDF of the distance between a random BNp and its altered version.** CDF of the distance between a random BNp and its altered version. (A) 6-gene networks for different m (number of mutations) and *p* (perturbation probability). (B) 6-gene networks with *m* = 2 mutations for different perturbation probabilities. (C) 8-gene networks with different m and *p*. (D) 8-gene networks with *m* = 2 mutations for different perturbation probabilities.

The estimated CDFs are shown in Figure 2, for several different parameters. Figure 2-(A) shows the estimation results for 6-gene networks for one or two gene mutations. We can see that the distance increases when we increase the number of mutations while keeping the probability of perturbation fixed. Similar behavior can be observed for 8-gene networks shown in Figure 2-(C). We can also see that the distance is generally larger for the 8-gene networks.

As we can see from Figure 2-(B), for 6-gene networks, increasing the perturbation probability from *p* = 0.001 to *p* = 0.01 decreases the distance. This is intuitive, since gene mutation (see (5)) only affects the regulatory part, which plays less important roles as the perturbation probability *p* increases. As a result, changing the regulatory structure of a BNp will have less significant effects when *p* is larger. Figure 2-(D) shows the results for 8-gene networks, which show similar tendencies.

### Performance of the algorithm on synthetic network families

We evaluated the performance of the proposed algorithm based on randomly generated families of Boolean networks through Monte Carlo simulations. All random networks in each of these families have identical structural properties (i.e., *p_b_* and *k*). In each family, one network whose Boolean functions are *canalizing* functions was selected as the true normal network *N_normal_*. A canalizing Boolean function is a function in which an input with a specific value determines the output of the function regardless of the other inputs. For instance, *f*(*x*_1_, *x*_2_) = *x*_1_O*Rx*_2_ is a canalizing function, where *x*_1_ = 1 (and similarly, *x*_2_ = 1) will make the output *f*(*x*_1_,*x*_2_) = 1, regardless of the value of the other input variable. We randomly chose a path of length *M* and altered the normal network according to the given path to obtain the cancerous network. The steady state distribution (SSD) of the cancerous network was computed, to be used as an input for the proposed algorithm. Next, we used the proposed algorithm to find out whether it was able to infer the true normal network from a given family of networks and correctly predict the actual cancer progression path, when provided with the number of mutations *M* and the cancerous SSD. In our simulations, we used *p_cancer_* = 0.001, *pb* = 0.3, and *k* = 2. We considered 6-gene networks with *M* = 2 mutations and 8-gene networks with *M* = 3 mutations. For the case of 6-gene networks, we considered families of size  and . For the case of 8-gene networks, we considered families of size . The algorithm was implemented using MATLAB 7.9.0 (R2009b), and all simulations have been performed on a desktop computer with 2.67GHz Intel Core i7 CPU and 12GB RAM. Each SSD computation took around 9.2 × 10^–4^ sec and 5.7 × 10^–3^ sec for *n* = 6 and *n* = 8, respectively.

Table 1 summarizes the results of applying our algorithm to 500 randomly generated network families, where each family contains  6-gene networks and the normal network undergoes two gene mutations. The threshold *β*_1_ was chosen such that  for different values of *p*_1_. The second column in Table 1 shows the probability of missing the true network defined in (16). The third, fourth, and fifth columns show the empirically estimated probabilities. The sixth column shows the average number of networks in the final network family . The seventh column shows the average number of cancerous paths found in the final step, and the final column shows the average number of SSD computations needed for finding . As we can see in Table 1, increasing *β*_1_ (by controlling *p*_1_) decreases the probability of missing the true normal network but increases the number of networks (and the corresponding cancerous paths) included in the final network family . Furthermore, the number of SSD computations will increase if we use a larger *β*_1_ (by increasing *p*_1_). A similar trend can be also observed in Table 2, which summarizes the simulation results for 200 families with  6-gene networks.

**Table 1:** Performance of the proposed algorithm evaluated on 500 randomly generated network families.

, *p_cancer_* = *p* = 0.001, *β*_2_ = 0.1							
*p*_1_	*P_miss_*				AVG of	AVG of # of paths	AVG of # of SSD calculations

*p*_1_ = 0.1	0.81	0.66	0.58	0.57	24.3	3.71	3,421
*p*_1_ = 0.3	0.49	0.45	0.41	0.39	45.11	4.17	4,677
*p*_1_ = 0.5	0.25	0.24	0.21	0.20	64.27	4.41	5,918
*p*_1_ = 0.7	0.09	0.06	0.04	0.04	94.84	4.60	9,208

Performance of the proposed algorithm evaluated on 500 randomly generated network families. Each family contained  6-gene networks (*k* = 2, *M* = 2, and *p_b_* = 0.3)

**Table 2:** Performance of the proposed algorithm evaluated on 200 randomly generated network families.

, *p_cancer_* = *p* = 0.001, *β*_2_ = 0.1							
*p*_1_	*P_miss_*				AVG of	AVG of # of paths	AVG of # of SSD calculations

*p*_1_ = 0.1	0.81	0.65	0.57	0.56	68.3	4.45	13,289
*p*_1_ = 0.3	0.49	0.46	0.40	0.39	138.17	4.70	17,319
*p*_1_ = 0.5	0.25	0.23	0.17	0.17	206.7	4.97	21,846
*p*_1_ = 0.7	0.09	0.05	0.03	0.03	293.4	4.98	36,348

Performance of the proposed algorithm evaluated on 200 randomly generated network families. Each family contained , 6-gene networks (*k* = 2, *M* = 2, and *p_b_* = 0.3).

We also evaluated the performance of the proposed algorithm based on randomly generated network families, each of which contains  networks with 8-genes, with *M* = 3 gene mutations. The experimental results are summarized in Table 3. The first column in this table shows the probabilities *p*_1_ and *p*_2_ that were used to choose *β*_1_ and *β*_2_, using (14). The threshold *β*_3_ was set to *β*_3_ = 0.1. Table 3 shows that increasing the threshold values result in a higher probability of success (i.e., smaller probability of missing the true normal network) but also a higher computational cost, as we would expect. In practical situations, the actual perturbation probability *p_cancer_* may not be exactly known, in which case we would have to estimate the probability. To evaluate the robustness of the proposed algorithm, in the presence of model mismatch, we performed another set of simulations, whose results are summarized in Table 4. We used randomly generated network families, each with  6-gene networks, and considered *M* = 2 mutations. As we can see in Table 4, there was no significant performance degradation when the perturbation probability *p* used by the algorithm was different from the true perturbation probability *p_cancer_*. The results for families with  networks are summarized in Table 5, which show that the proposed algorithm is robust to model mismatch. Finally, Table 6 shows the results for network families with  8-gene networks with *M* = 3 gene mutations, which also clearly shows the robustness of our algorithm.

**Table 3:** Performance of the proposed algorithm evaluated on 100 randomly generated network families

, *p_cancer_* = *p* = 0.001, *β*_3_ = 0.1							
*p*_1_, *p*_2_	*P_miss_*				AVG of	AVG of # of paths	AVG of # of SSD calculations

*p*_1_ = 0.1, *p*_2_ = 0.1	0.95	0.74	0.68	0.68	28.6	8.74	8,158
*p*_1_ = 0.3, *p*_2_ = 0.3	0.66	0.42	0.36	0.34	57.5	10.2	13,999
*p*_1_ = 0.5, *p*_2_ = 0.5	0.34	0.16	0.13	0.13	111.3	13.04	35,039
*p*_1_ = 0.7, *p*_2_ = 0.7	0.11	0.05	0.03	0.03	123.1	11.45	89,788

Performance of the proposed algorithm evaluated on 100 randomly generated network families. Each family contained  8-gene networks (*k* = 2, *M* = 3, and *p_b_* = 0.3)

**Table 4:** Performance of the proposed algorithm in case of model mismatch. Evaluated on 500 randomly generated network families.

, *p_cancer_* = 0.001, *p* = 0.003, *β*_2_ = 0.1						
*p*_1_				AVG of	AVG of # of paths	AVG of # of SSD calculations

*p*_1_ = 0.1	0.88	0.77	0.76	8.65	2.3	3177.1
*p*_1_ = 0.3	0.49	0.43	0.42	46.8	4.29	4693.9
*p*_1_ = 0.5	0.25	0.20	0.19	61.31	4.35	5802.1
*p*_1_ = 0.7	0.18	0.15	0.14	77.82	4.41	6870.7

Performance of the proposed algorithm in case of model mismatch. Evaluated on 500 randomly generated network families. Each family contained  6-gene networks (*k* = 2, *M* = 2, and *p_b_* = 0.3).

**Table 5:** Performance of the proposed algorithm in case of model mismatch. Evaluated on 200 randomly generated network families.

, *p_cancer_* = 0.001, *p* = 0.003, *β*_2_ = 0.1						
*p*_1_				AVG of	AVG of # of paths	AVG of # of SSD calculations

*p*_1_ = 0.1	0.94	0.82	0.80	14.33	2.52	12,454
*p*_1_ = 0.3	0.43	0.37	0.36	140.5	4.9	17,850
*p*_1_ = 0.5	0.28	0.22	0.21	172.48	4.54	21,175
*p*_1_ = 0.7	0.19	0.13	0.13	234.8	4.60	26,060

Performance of the proposed algorithm in case of model mismatch. Evaluated on 200 randomly generated network families. Each family contained , 024 6-gene networks (*k* = 2, *M* = 2, and *p_b_* = 0.3).

**Table 6:** Performance of the proposed algorithm in case of model mismatch. Evaluated on 100 randomly generated network families.

, *p_cancer_* = 0.001, *p* = 0.003, *β*_3_ = 0.1						
*p*_1_,*p*_2_				AVG of	AVG of # of paths	AVG of # of SSD calculations

*p*_1_ = 0.1, *p*_2_ = 0.1	0.95	0.76	0.75	25.75	7.88	5,724
*p*_1_ = 0.3, *p*_2_ = 0.3	0.48	0.44	0.43	46	10.79	12,720
*p*_1_ = 0.5, *p*_2_ = 0.5	0.21	0.17	0.16	97.8	14.34	30,146
*p*_1_ = 0.7, *p*_2_ = 0.7	0.19	0.14	0.14	100.8	9.69	47,755

Performance of the proposed algorithm in case of model mismatch. Evaluated on 100 randomly generated network families. Each family contained  8-gene networks (*k* = 2, *M* = 3, and *p_b_* = 0.3).

### Performance on cancerous networks involving the p53 gene

**Figure 3:**
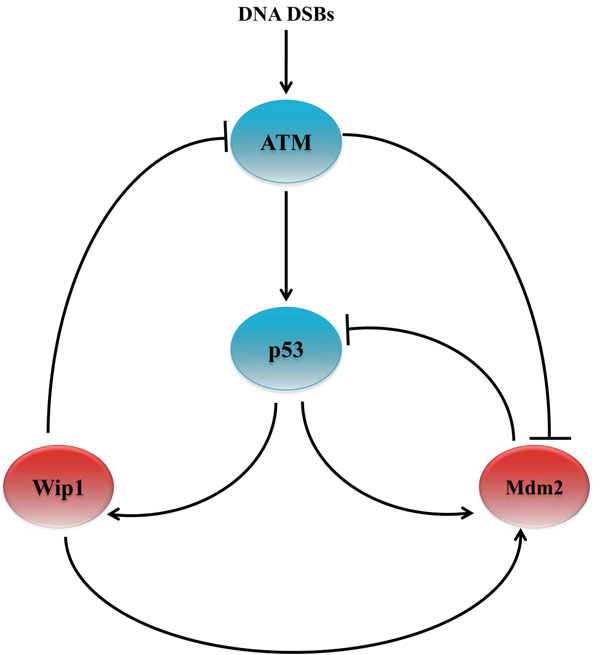
ATM–p53–Wip1–Mdm2 pathways

Next, we evaluated the performance of the proposed algorithm based on a family of BNs constructed from pathways that involve the *p*53 gene. Tumor suppressor gene *p*53 has been extensively studied and it is known to be involved in various well-known biological pathways. It has been observed that *p*53 is mutated in 30-50% of common human cancers [3]. In fact, in the presence of DNA damage, a mutant *p*53 may lead to the emergence of abnormal cells. Figure 3 shows the ATM-p53-Wip1–Mdm2 pathways that involve the tumor suppressor gene p53 [18].

**Figure 4:**
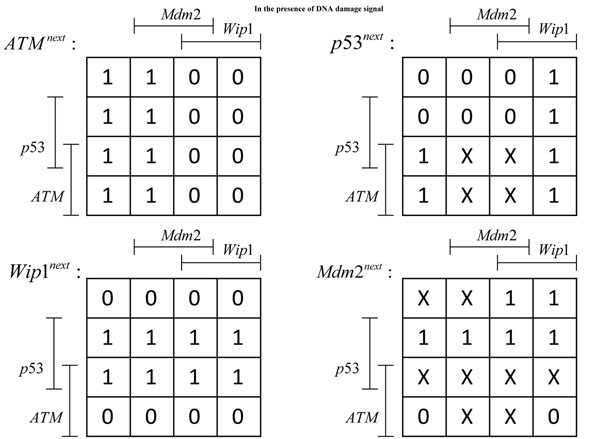
**Karnaugh maps generated by the pathways shown in Figure**3

These pathways operate in different ways depending on the context, determined by the presence (or absence) of a DNA damage event that results in DNA double-strand breaks. Here, we consider the case when DNA damage is present, which may lead to the development and progression of tumor. Under this context, we consider single and double mutations in the given pathways, where we focus on the mutation of *p*53 and *Mdm*2. Each gene alteration can be one of the three forms: *mutation*, *amplification*, or *deletion*. Sequencing data of 138 patients with glioblastoma, provided by TCGA, showed that 32% and 12% of the patients suffered from the alteration in the *p*53 and *Mdm*2 genes, respectively. Also among 316 patients with serous ovarian cancer, 96% suffered from the mutation of *p*53. A similar study has revealed that about 26% of 216 patients with sarcoma have amplified *Mdm*2. Mutation in *p*53 and amplification in *Mdm*2 have been also simultaneously observed in some cases. Based on these observations made in existing cancer studies, we consider the following types of alterations in our experiments: (*p*53,0), (*Mdm*2,1), and {(*p*53, 0), (*Mdm*2, 1)}, where *p*53 is permanently deactivated and/or *Mdm*2 is permanently activated. In a recent work [1], it has been shown that the pathways in Figure 3 do not uniquely determine the normal Boolean network *N_normal_* that governs healthy cells. We used the method proposed in [1] to enumerate all possible Boolean networks that satisfy the constraints imposed by the given pathways. Following [1], we constructed four *Karnaugh maps*, one for each gene in the given pathways. Karnaugh maps have been used in logic circuit design to simplify a given Boolean function and derive its minimal representation. In a Karnaugh Map [19], each position in the map (i.e., an element in a matrix) corresponds to a specific state (defined by the values of all genes in the network), such that neighboring states have unit Hamming distance. The value at each position indicates the value of a particular gene at the next time point, which is a Boolean function of the current state. The resulting maps are shown in Figure 4. In these tables, each line-segment, attached to a gene, shows the locations where that gene the takes value 1. The symbol *X* is used to indicate positions where the available pathway information was not enough for uniquely determining the table entries. These entries may take either 0 or 1 without violating the constraints. As a result, the given Karnaugh maps give rise to an uncertainty class of networks  that contains 2^12^, where 12 is the number of entries in the given maps that cannot be uniquely determined. Since *Mdm*2 is directly connected to three genes in Figure 3, we assume the connectivity to be *k* = 3, which is used to estimate the CDF of the distance between a random BNp and its altered version. The BN reported in [1] is assumed to be the true normal network *N_normal_*, as this network was shown to faithfully reproduce the experimentally observed behavior of the genes in published literature. We assumed *p_cancer_* to be 0.001. As in the previous section, we evaluated the performance of the proposed algorithm under two different cases: when we have a perfect estimate of the perturbation probability (*p* = *p_cancer_*) and when there is a model mismatch (*p* ≠ *p_cancer_*).

### Case-1: deactiviation of p53

We considered the alteration of the Boolean network reported in [1] through the permanent deactivation of *p*53 (i.e. (*p*53, 0)). We used our algorithm to detect the true normal network and the gene mutation. Table 7 shows the simulation result when the threshold was set to *β*_1_ = 0.05. The second column in the table shows the number of networks in the final network family, and the third column shows the total number of network-path pairs predicted by the algorithm. The fourth column shows the number of different paths in the predicted result. We also categorized the result of each experiment as a “success (S)” or a “failure (F)”, based on whether the final prediction contained the true network-path pair or not. As we can see, our algorithm was able to reduce the uncertainty class of networks without missing the true network for *p* = 0.001,0.003,0.005. For *p* = 0.007, the algorithm missed the true network, mainly because the perturbation probability was high enough to render the effects of the regulatory structure of the network relatively insignificant. Increasing *β*_1_ from 0.05 to 0.1 increases the number of network-path pairs included in the final prediction, thereby preventing the algorithm from missing the true network, as shown in Table 8. In terms of fault detection, the proposed algorithm performed very well. As shown in Table 7 and Table 8, the algorithm was able to correctly pinpoint the actual gene mutation out of 8 possible mutations, when it was successful.

**Table 7:** Performance of the proposed algorithm in the case when *p*53 is deactivated.

*p*	# networks	# network-path pairs	# paths	result
0.001	2048	2048	1	S
0.003	2048	2048	1	S
0.005	512	512	1	S
0.007	0	0	0	F

Performance of the proposed algorithm in the case when *p*53 is deactivated. Threshold was set to *β*_1_ = 0.05 and the true perturbation probability was assumed to be (*p_cancer_* = 0.001).

**Table 8:** Performance of the proposed algorithm in the case when *p*53 is deactivated.

*p*	# networks	# network-path pairs	# paths	result
0.001	2048	2048	1	S
0.003	2048	2048	1	S
0.005	1904	1904	1	S
0.007	832	832	1	S

Performance of the proposed algorithm in the case when *p*53 is deactivated. Threshold was set to *β*_1_ = 0.1 and the true perturbation probability was assumed to be (*p_cancer_* = 0.001).

### Case-2: amplification of Mdm2

Next, we altered the normal network by mutating *Mdm2* such that it is amplified (i.e. (*Mdm*2,1)). The results are summarized in Table 9 and Table 10 for *β*_1_ = 0.05 and *β*_1_ = 0.1, respectively. For *β*_1_, our algorithm was able to reduce the uncertainty class of networks without missing the true normal network for *p* = 0.001 and *p* = 0.003. When the perturbation probability became larger, the regulatory structure from the pathways was obscured, and the algorithm was not able to effectively reduce the uncertainty class (e.g., see Table 9, *p* = 0.005 and *p* = 0.007). By increasing *β*_1_ from 0.05 to 0.1, the algorithm could successfully reduce the uncertainty class for *p* = 0.005, as shown in Table 10.

**Table 9:** Performance of the proposed algorithm in the case when *Mdm*2 is amplified.

*p*	# networks	# network-path pairs	# paths	result
0.001	1088	1174	3	S
0.003	520	540	2	S
0.005	0	0	0	F
0.007	0	0	0	F

Performance of the proposed algorithm in the case when *Mdm*2 is amplified. Threshold was set to *β*_1_ = 0.05 and the true perturbation probability was assumed to be (*p_cancer_* = 0.001).

**Table 10:** Performance of the proposed algorithm in the case when *Mdm*2 is amplified.

*p*	# networks	# network-path pairs	# paths	result
0.001	1088	1184	3	S
0.003	832	894	3	S
0.005	520	544	2	S
0.007	0	0	0	F

Performance of the proposed algorithm in the case when *Mdm*2 is amplified. Threshold was set to *β*_1_ =0.10 and the true perturbation probability was assumed to be (*p_cancer_* = 0.001).

### Case-3: simultaneous deactivation of p53 and amplification of Mdm2

Finally, we considered the case when *p*53 was deactivated and *Mdm*2 was amplified at the same time. Table 11 and Table 12 summarize the results of applying the proposed algorithm for the case of double gene mutations: (*p*53,0) and (*Mdm*2,1). As we would expect, the proposed algorithm did not perform well in this case, since introducing two gene mutations in a 4-gene network almost completely obscures the regulatory structure in the original normal network. In fact, the networks in the initial uncertainty class  will yield similar (or identical) SSDs once we mutate two genes. These results lead to an interesting insight into the expected performance of the proposed algorithm. As mentioned throughout the paper, the proposed algorithm aims to backtrace the set of gene mutations that has led to an (unknown) cancerous gene regulatory network with a given SSD. Suppose the number of mutations *M* is relatively small compared to the total number of genes *n* in the network (e.g., *M*/*n* ≈ 0). In such a case, the dynamics of the cancerous network would be largely governed by the regulatory mechanisms in the original healthy network. Even though it is theoretically possible that a few gene alterations lead to significant changes in the overall SSD, identifying these alterations would be still feasible since the regulatory structure of the original network would remain mostly intact. However, if the number of mutations gets larger (e.g., *M*/*n* ≈ 1), the activity of many genes would be “frozen”, either being permanently deactivated or permanently amplified, in which case the dynamics and the regulatory structure of the original network would be significantly lost. As a result, networks that originally have very distinct structures may yield similar SSDs as a result of the accrued mutations. In this case, it would be difficult for the algorithm to make predictions with high accuracy, since the available information would be too small to effectively cope with the present uncertainty.

**Table 11:** Performance of the proposed algorithm when *p*53 is deactivated and *Mdm*2 is amplified.

*p*_1_	# networks	# network-path pairs	# paths	result	# SSD calculations
*p*_1_ = 0.1	730	1333	4	F	38,684
*p*_1_ = 0.3	2458	3810	4	F	69,050
*p*_1_ = 0.5	3937	7208	4	S	93,650
*p*_1_ = 0.7	4096	9009	4	S	113,612

Performance of the proposed algorithm when *p*53 is deactivated and *Mdm*2 is amplified. Several different values of *β*_1_ was used (by varying *p*_1_), and *β*_2_ was set to 0.1. The perturbation probability was assumed to be known (*p* = *p_cancer_* = 0.001).

**Table 12:** Performance of the proposed algorithm when *p*53 is deactivated and *Mdm*2 is amplified.

*p*_1_	# networks	# network-path pairs	# paths	result	# SSD calculations
*p*_1_ = 0.1	1063	1629	4	F	40,016
*p*_1_ = 0.3	1661	2582	4	F	48,038
*p*_1_ = 0.5	2704	4089	4	F	69,146
*p*_1_ = 0.7	4096	8839	4	S	101,426

Performance of the proposed algorithm when *p*53 is deactivated and *Mdm*2 is amplified. Several different values of *β*_1_ was used (by varying *p*_1_), and *β*_2_ was set to 0.1. We assumed that the true perturbation probability is unknown, hence there is a model mismatch (*p* = 0.003, *p_cancer_* = 0.001).

## Conclusions

We proposed an effective probabilistic algorithm for reconstructing the tumor progression process. Given an uncertainty class of networks, which arises from our partial knowledge of the true gene regulatory network represented by biological pathways, and the steady state distribution of a cancerous network, the proposed algorithm tries to simultaneously infer the true gene regulatory network that underlies healthy cells and to predict the sequence of gene mutations that occurred during the tumor progression process. As demonstrated by our experiments, based on both randomly generated networks and realistic networks constructed from known biological pathways that involve the tumor suppressor gene *p*53, our algorithm can effectively cope with the uncertainty present in gene regulatory networks and accurately infer the normal (healthy) network and the actual path of tumor progression with high probability. Furthermore, the proposed algorithm is robust to model mismatch and provides us with effective means for trading prediction accuracy for computational efficiency.

The computational complexity of the algorithm depends on the number of genes in the network, the number of mutations, and the number of networks in the initial uncertainty class, and increasing any of these numbers will increase the computational overhead. Based on the mathematical representation of Boolean networks, increasing the number of genes will exponentially increase the number of possible networks. However, this rapid increase does not necessarily mean that the size of the uncertainty class of networks that we need to deal with will increase at the same rate. For example, many of the mathematically possible networks may not be considered biologically viable, hence may be omitted in practice. Moreover, although the total number of states in a Boolean network with *n* genes is 2*^n^*, many states may be eliminated via state reduction, and the reduced network may consist of considerably fewer states [20]. In fact, the whole idea of network reduction is relevant to the present problem, just as it is to determining control polices for gene regulatory networks, where computational intractability prohibits the design of control policies without constraining the network size [21]. For example, even when the gene expression levels are restricted to be binary, a network with 15 genes, absent some form of state reduction, cannot be considered, because the size of the resulting transition probability matrix would be 2^15^ × 2^15^, making any kind of dynamic or control analysis intractable. Another possible way to reduce the complexity of the algorithm is to restrict the possible gene mutations via the use of prior knowledge. For example, we may restrict the possible mutant genes only to a smaller subset of genes that are known to be susceptible to mutation. Furthermore, prior knowledge concerning the expected type of mutation for a susceptible gene (e.g., “amplification” for oncogenes and “deactivation” for tumor suppressor genes) can be taken into account. Although we did not constrain the possible gene mutations nor applied any network reduction technique in this study, such modifications are fairly straightforward and may be used to enhance the overall computational efficiency of the proposed algorithm.

## Authors' contributions

Conceived and designed the experiments: MSE, ERD. Developed the algorithm and performed the experiments: MSE. Analyzed the data: MSE, ERD, BJY. Wrote the paper: MSE, ERD, BJY.

## Competing interests

The authors declare that they have no competing interests.

## Supplementary Material

Additional file 1 Click here for file

## References

[B1] Layek R Datta A Dougherty E From biological pathways to regulatory networks Mol. BioSyst 2011 7 843 851 10.1039/c0mb00263a 21161088

[B2] Layek R Datta A Bittner M Dougherty E Cancer therapy design based on pathway logic Bioinformatics 2011 27 4 548 10.1093/bioinformatics/btq703 21193523

[B3] Weinberg R The biology of cancer 2007 Garland Science New York

[B4] Gerstung M Baudis M Moch H Beerenwinkel N Quantifying cancer progression with conjunctive Bayesian networks Bioinformatics 2009 25 21 2809 10.1093/bioinformatics/btp505 19692554 PMC2781752

[B5] Kauffman S The origins of order: Self organization and selection in evolution 1993 Oxford University Press, USA

[B6] Aldana M Coppersmith S Kadanoff L Boolean dynamics with random couplings Perspectives and Problems in Nonlinear Science 2003 23 89

[B7] Serra R Villani M Semeria A Genetic network models and statistical properties of gene expression data in knock-out experiments Journal of theoretical biology 2004 227 149 157 10.1016/j.jtbi.2003.10.018 14969713

[B8] Dougherty E Pal R Qian X Bittner M Datta A Stationary and structural control in gene regulatory networks: basic concepts International Journal of Systems Science 2010 41 5 16 10.1080/00207720903144560

[B9] Qian X Dougherty E On the long-run sensitivity of probabilistic Boolean networks Journal of theoretical biology 2009 257 4 560 577 10.1016/j.jtbi.2008.12.023 19168076 PMC2660388

[B10] Marshall S Yu L Xiao Y Dougherty E Inference of a probabilistic Boolean network from a single observed temporal sequence EURASIP Journal on Bioinformatics and Systems Biology 2007 2007 5 10.1155/2007/32454 PMC3171335 18364987

[B11] Ivanov I Simeonov P Ghaffari N Qian X Dougherty E Selection policy-induced reduction mappings for Boolean networks Signal Processing, IEEE Transactions 2010 58 9 4871 4882

[B12] Hunter J Stationary distributions and mean first passage times of perturbed Markov chains Linear Algebra and its Applications 2005 410 217 243

[B13] Schweitzer P Perturbation theory and finite Markov chains Journal of Applied Probability 1968 5 2 401 413 10.2307/3212261

[B14] Qian X Dougherty E Effect of function perturbation on the steady-state distribution of genetic regulatory networks: optimal structural intervention IEEE Trans. Signal Process 2008 56 10-1 4966 4976

[B15] Kauffman S Peterson C Samuelsson B Troein C Genetic networks with canalyzing Boolean rules are always stable Proceedings of the National Academy of Sciences of the United States of America 2004 101 49 17102 10.1073/pnas.0407783101 15572453 PMC534611

[B16] Shmulevich I Dougherty E Genomic Signal Processing (Princeton Series in Applied Mathematics) 2007 Princeton University Press

[B17] Shmulevich I Lahdesmaki H Dougherty E Astola J Zhang W The role of certain Post classes in Boolean network models of genetic networks Proceedings of the National Academy of Sciences of the United States of America 2003 100 19 10734 10.1073/pnas.1534782100 12963822 PMC202352

[B18] Batchelor E Loewer A Lahav G The ups and downs of p53: understanding protein dynamics in single cells Nature Reviews Cancer 2009 9 5 371 377 10.1038/nrc2604 19360021 PMC2892289

[B19] Karnaugh M The map method for synthesis of combinational logic circuits Trans. AIEE. pt. I 1953 72 9 593 599

[B20] Qian X Ghaffari N Ivanov I Dougherty E State reduction for network intervention in probabilistic Boolean networks Bioinformatics 2010 26 24 3098 10.1093/bioinformatics/btq575 20956246 PMC3025721

[B21] Ghaffari N Ivanov I Qian X Dougherty E A CoD-based reduction algorithm for designing stationary control policies on Boolean networks Bioinformatics 2010 26 12 1556 10.1093/bioinformatics/btq225 20421196

